# Certificates to the Efficacy of Secret Remedies

**Published:** 1855-09

**Authors:** 


*Certificates to the efficacy of secret remedies.*—There are few ways in which good-natured people are doing so much harm, while meaning to do good, as in signing their names to medical certificates, to be distributed all over the land in the public newspapers, or thrust upon us, whether we will or no, at our very doors. This has got to be a crying evil in the land, and it is time it was corrected. It is doing much, very much, to impair the faith in remedies properly given, to shake confidence in the medical profession, and thus incidentally to re-act upon the community to their great disadvantage. It is a gross injury to the medical profession. It constantly taunts them with the insinuation, "Look here! what all your boasted science could not do, this man whom you denounce as an ignoramus, has done! This medicine has cured me; yours did me no good, and I might have been in my grave for all you!

From this time forth, fling doctor's physic to the dogs, and let me have only the healing drugs of the inspired natural physician!" Waiving for a moment all question as to the amount of real efficacy in the remedies thus vaunted—granting even, for argument's sake, that the facts are as stated, what does it amount to? Only that a drug taken at the right moment has wrought a good effect, no more. For this shall it be emblazoned in every newspaper sheet as the great catholicon? Why, there are two hundred physicians in Boston, doing, with the blessing of Providence, precisely the same thing every day! And are they justified in proclaiming their skill and the power of their remedies for this reason? No true physician, at any rate, would give an affirmative answer. And yet their patients forsake them, or secretly use while under their care any nostrum their assiduous friends recommend. Or if they ask the advice of their physician as to the expediency of trying the remedy, he has no means of knowing its real character, and of course cannot recommend it. Still human nature is such that even the mystery which shrouds it gives it a fascination, particularly if the dose be *pleasant to the taste.* Men of respectable position in the community give up their business to devote themselves to the sale of the article, and untold sums are their reward. Grateful patients eagerly subscribe the certificate of its real or supposed virtues, while yet in the flush of their enthusiasm at its efficacy, and the document goes to the world, to add one more to the legion of medical delusions which are a stigma upon the present generation. Let any intelligent man go into a public hospital, and he will find numbers of patients who will tell even more extraordinary experiences of their own—how they were full of pain, motionless, helpless, but in twenty-four hours they were changed men, to all intents and purposes cured. Others will tell you that for years they had not had the use of their limbs, but now they walk rejoicing. Happy as these occurrences are, blessed indeed to the patients, the physician who has been the fortunate instrument in bringing them about does not, or ought not, to claim any exclusive merit; rather does he most willingly point out to his professional brethren the means he has used with such success. Thus is the blessing extended beyond his own immediate sphere, and no selfish thought of personal profit limits the exercise of his professional benevolence. The medical journals are crowded with reports of just such cases, which go forth over the whole world to bless mankind. Nay, *these very remedies* are not unfrequently taken up by shrewd nostrum venders, and after being disguised by some inert addition, are sold to the unsuspecting public at a profit of thousands per cent., without the least credit being given to the man who first applied it for such a purpose. But there is another very important consideration which is entirely overlooked by the patent-medicine-swallowing, or the puffed-medicine-swallowing community, which is this—*medicines positively effective for good are equally powerful for evil.* The knife is a catlin with two edges, let him who uses it see that he cuts not his own fingers. And this is no imaginary casualty. It is a fact well known to physicians, that cases are frequently brought under their own observation, of excellent remedies having been taken to great excess when not administered by the direction of a professional man. It is astonishing to see the recklessness with which people sometimes do this, taking *ad libitum* the most powerful medicines of the pharmacopœia, simply because under some circumstances they have been recommended to them by some respectable physician, who never intended they should be adopted into such promiscuous use. The charity of these good people is most liberally extended to their friends, inducing them to take the same, they alone being responsible for the consequences. And the reason for this—not always, but often—is to avoid the necessity of paying the physician the fee which is his due for advice on the subject. Again, supposing the the secret remedy to have been effectual in certain cases, *the cases in which it has failed or done harm* far out-number them, and of these the community hears not. Many of the cases of supposed cure turn out to be only transiently benefited, and the deluded sufferer only falls back again into greater hopelessness. Mean-while the certificate stands, and money pours into the pocket of the nostrum-vender—what else does he care for?

We would remark in conclusion that—medical discoveries should be the property of the human race; as free for the use of mankind as heaven's natural gifts of light and air. Only when open to such unrestricted use can their real value be tested. The noble calling of physician ought not to be degraded to a mere matter of making money; the community are as much interested in this as physicians, perhaps more so. By following the course which is getting to be so common, they are discouraging and starving those whom they ought to regard as their true friends.—*Boston Medical and Surgical Journal.*

